# Indexing Arbitrary-Length *k*-Mers in Sequencing Reads

**DOI:** 10.1371/journal.pone.0133198

**Published:** 2015-07-16

**Authors:** Tomasz Kowalski, Szymon Grabowski, Sebastian Deorowicz

**Affiliations:** 1 Institute of Applied Computer Science, Lodz University of Technology, Al. Politechniki 11, 90-924 Łódź, Poland; 2 Institute of Informatics, Silesian University of Technology, Akademicka 16, 44-100 Gliwice, Poland; University of Maryland, UNITED STATES

## Abstract

We propose a lightweight data structure for indexing and querying collections of NGS reads data in main memory. The data structure supports the interface proposed in the pioneering work by Philippe et al. for counting and locating *k*-mers in sequencing reads. Our solution, PgSA (pseudogenome suffix array), based on finding overlapping reads, is competitive to the existing algorithms in the space use, query times, or both. The main applications of our index include variant calling, error correction and analysis of reads from RNA-seq experiments.

## Introduction

The genome sequencing costs dropped recently to less than 5 thousand U.S. dollars per human genome with about 30-fold coverage [1]. Using the recent (and expensive) Illumina HiSeq X Ten system [2], it may be even possible to reduce this cost to about 1 thousand dollars (or somewhat more) on a long run. The scale of the largest sequencing projects is amazing, e.g., the Million Veteran Program [3] aims at sequencing 1M human genomes. Needless to say, all this results in enormous amounts of sequencing data.

These data have to be processed in some way. Usually, they are mapped onto reference genomes and then variant calling algorithms are used to identify the mutations present in sequenced genomes. Since the mapping requires fast search over reference genomes, a lot of indexing structures for genomes were adopted or invented. The obvious candidates were the suffix tree and the suffix array [4], but their space requirements were often prohibitive, especially in the beginning of the 21st century. The situation changed with the advent of much more compact (compressed) index data structures. The most widely used in the read aligning software is the family of FM-indexes [5], employed by the popular Bowtie [6], BWA [7] and many other mappers. Modern computers are more powerful, hence nowadays using a suffix array is often not a problem, especially if the array is sparsified (i.e., only a fraction of indexes is represented explicitly) [8]. One of the recent successful examples is the MuGI multi-genome index [9], allowing to index 1092 human genomes in less than 10 GB of memory.

As said above, a lot was done in the area of genome indexing, but very little for the other standard component of the input, i.e., sequencing reads. The main reason is that when the reads are simply mapped onto a reference genome, indexing them is pointless. In many situations, however, the reads are processed in some way before (or instead of) mapping. The most obvious case is read correction, which makes the mapping (or de novo assembling) easier and yields better final results, i.e., better determination of mutations. There are a number of read correctors, e.g., Quake [10], RACER [11], BLESS [12], Fiona [13]; see the recent survey [14] for more examples. Sometimes the paired reads are joined if they overlap, with benefits in the mapping quality [15]. In some other applications, e.g., in metagenomic studies, the goal is to assign reads to species (to identify which organisms can be found in the analyzed probe), and the reads are not mapped at all [16–18].

In such cases no reference sequence is used (or it is used only implicitly) and all the available knowledge can be retrieved only from the reads. The simplest approach is to calculate the statistics of *k*-mers (i.e., all *k*-symbol long substrings of reads), but some programs use more sophisticated knowledge. Therefore, the necessity of indexing reads was identified recently [19]. Philippe et al. defined therein the index supporting the following queries. Given a query string *f*:

*Q*1 In which reads does *f* occur?
*Q*2 In how many reads does *f* occur?
*Q*3 What are the occurrence positions of *f*?
*Q*4 What is the number of occurrences of *f*?
*Q*5 In which reads does *f* occur only once?
*Q*6 In how many reads does *f* occur only once?
*Q*7 What are the occurrence positions of *f* in the reads where it occurs only once?

There are two ways in which *f* can be given in those queries, which may lead to different time complexities and actual timing results. In one, *f* is given as a sequence of DNA symbols. In the other, *f* is represented as a read ID followed with the start position of *f* in this read (and optionally, *f*’s length, if it is not fixed).

There are a number of potential applications of this index. Philippe et al. [19] described the following. The queries *Q*1 and *Q*2 can be used for mutation (both SNPs and short indels) detection. The query *Q*2 can be used to calculate a “local coverage” of a *k*-mer, i.e., the number of reads sharing it. This was used in the work [20] for calculation of “support profile” of each *k*-mer in a large package for analyzing reads from RNA-seq experiments. One more potential usage of index queries *Q*3 and *Q*4 can be in clustering and assembly without a reference genome.

One of the successful techniques in read correctors, e.g., BLESS, RACER, is to preprocess the reads to collect the *k*-mer frequencies (i.e., allow to answer the *Q*4 queries), which can be obtained with specialized software [21–23]. In some other tools, like Fiona [13], Shrec [24], HybridShrec [25], it is necessary also to obtain the list of reads containing the *k*-mer (i.e., they need *Q*1 queries). The solution used in Fiona is to construct the generalized suffix array, i.e., suffix array containing all suffixes from all reads. Unfortunately, this implies huge memory requirements, e.g., for reads of human sequencing with 10-fold coverage, the memory occupation is 224 GB.

The recent paper by Salzberg et al. [26] deals with mutation detection. One of the main difficulties in this problem is a large amount of candidate mutations that must be filtered out. Salzberg et al. propose an innovative approach in their Diamund software. At the first stages, they collect the statistics of *k*-mers in the sequencing results of a trio (mother, father, proband). Then the statistics are reduced by a huge factor in some way. More precisely, Diamund attempts to identify all *k*-mers unique to an affected proband and missing from both unaffected parents. The proband data are filtered to remove the *k*-mers likely to contain sequencing errors, based on the assumption that any *k*-mer occurring just a few times (in a dataset with a high coverage) represents an error. Intersecting all three sets identifies *k*-mers that are unique to the proband. Finally, when the number of different *k*-mers is counted in (tens of) thousands, they need to identify the reads containing these *k*-mers. Diamund uses Kraken [17] or MUMmer [27] for this task. Nevertheless, this is an obvious potential application of an index for sequencing reads.

Currently, only a few indexing structures supporting the mentioned list of queries are known. Historically, the first one is Gk arrays (GkA) [19]. This scheme works for a single length of *f* only (set at construction time). The main GkA idea is to order lexicographically all substrings of length *k* = ∣*f*∣ of the reads. Let us denote the cardinality of the reads collection with *q*. Assume that the reads are of equal length *m*. As the number of reads substrings is *q*(*m* − *k* + 1), the binary search for sequences with a given *k*-long prefix, like in a suffix array [28], has time complexity of *O*(*k* log((*m* − *k*+1)*q*)). In the following we use the symbol *n* = *q*(*m* − *k*+1) to simplify notation. This operation answers query *Q*4 with *f* given as a sequence of symbols. If, however, the query position is given, then *Q*4 is handled in constant time. GkA is based on three arrays: one for storing the start position of each *k*-mer, one inverted array telling the lexicographic rank of a *k*-mer given its position in a read, and finally an array associating to a *k*-mer’s rank its number of occurrences. The proposed data structure was found to be both more memory efficient and (in most cases) faster than two alternatives, a hash table and a suffix array augmented with some helper tables.

Välimäki and Rivals [29] proposed a compressed variant of Gk arrays, based on the compressed suffix array (CSA) [30]. Their index, CGkA, reduces the size of its predecessor by about 40% to 90%, handling most queries with similar time complexity. There is a sampling rate parameter in the CGkA index telling how many, evenly sampled, suffix array and inverted suffix array entries are stored directly. Like GkA, this solution also supports a single value of *k*.

The index presented in this paper is based on two ideas: building a pseudogenome by finding overlapping reads in the collection, and using the sparse suffix array [8] as the search engine in the resulting sequence. We performed a number of experiments to compare the proposed PgSA (pseudogenome suffix array) and the existing GkA and CGkA indexes for the supported queries. Then, to see how PgSA would work in a real environment, we replaced the GkA in CRAC [20] by our index to check its overall memory consumption and processing time.

## Materials and Methods

We assume that the input alphabet contains 4 (ACGT) or 5 symbols (ACGTN). The actual number of symbols in the input data implies some design choices in the internal representation of our index. By a *pseudogenome* we mean a sequence obtained by concatenation of all (possibly reverse-complemented) reads with overlaps. More formally, let us have a read array 𝓡 = [*R*_1_, …, *R*_*q*_], where *R*_*i*_ = *R*_*i*_[1…*m*] for all *i* ∈ {1, …, *q*}. A pseudogenome is a sequence *PG*[1…*p*] for which

- there exists a sequence *j*_1_, *j*_2_, …, *j*_*q*_ such that *j*_1_ = 1, *j*_*i*+1_ − *j*_*i*_ ∈ {0, 1, …, *m*} for all *i* ∈ {2, …, *q*} and *j*_*q*_ = *p* − *m* + 1,
- for each *j*_*i*_ we have *PG*[*j*_*i*_…*j*_*i*_ + *m* − 1] = *R*_*u*_*i*__ or *PG*[*j*_*i*_…*j*_*i*_+*m* − 1] = *rc*(*R*_*u*_*i*__), where *rc*(⋅) is the reverse-complement operation on a DNA sequence,
- [*u*_1_, *u*_2_, …, *u*_*q*_] is a permutation of {1, 2, …, *q*}.

We attempt to minimize the pseudogenome length *p*. In further considerations we usually deal with the permuted read array, hence we define it as $R^{\prime }=\left[R_{u_{1}},\ldots ,R_{u_{q}}\right]$, where the indices *u*_*i*_ are described just above. Additionally, two symbols, + and ∘, will be useful. *S*+*T* is a plain concatenation of strings *S* and *T*. *S* ∘ *T* denotes a concatenation of strings *S* and *T* with a non-zero overlap of maximal length.

While a sequence approximating a *real* genome may be obtained by a de novo assembly procedure, we refrain from it because of two reasons. First, our procedure is lightweight, at least in conceptual and programming sense, while the problem of de novo assembly is known to be hard. Second, removing sequencing errors during the assembly is obviously beneficial for the output accuracy, but we aim at indexing original reads, and mapping the reads onto a “corrected” genomic sequence would complicate the index representation and would possibly be detrimental to query handling effectiveness.

Note that the minimal pseudogenome problem, without allowing the reverse-complement operations on the reads, is known in string matching literature under the name of the shortest common superstring (SCS) problem. SCS is NP-hard, as shown by Maier and Storer [31].

**Fig 1:**
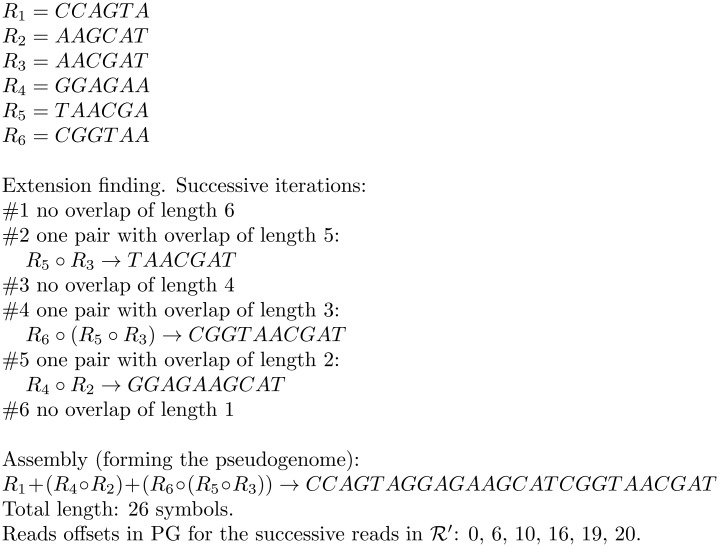
Pseudogenome generation example. The input read collection 𝓡 contains 6 reads of length 6.

The pseudogenome is generated in the following way. (Fig 1 illustrates the main idea of the construction algorithm.) We keep the reads packed, having 3 symbols (when *σ* = 5) or 4 symbols (when *σ* = 4) per byte. The alphabet size is found in a preliminary pass over the data. We will say that a read has a prefix (suffix) overlap if it is already preceded (followed) with another read with a non-empty overlap. During the main phase of the algorithm we maintain five main arrays: *P*, *Q*, *Q*′, *S*, and *S*′. The main loop of the algorithm is run *m* − 1 times. In each loop iteration, the following invariants are held:

- The elements of array *P* have two fields, the information if the current read (i.e., with the ID given by the current index in *P*) has a suffix overlap and if so, the ID of the suffix-overlapping read and the overlap length.
- Array *Q* always stores the IDs of the reads which are not suffix-overlapping any other reads. The items in *Q* are arranged by the lexicographical order of the reads.
- Array *S* always stores the IDs of the reads which are not prefix-overlapping any other reads. In *i*-th loop iteration, *i* ≥ 1, they are arranged by the lexicographical order of the suffix of the read starting at the position *i*.

At the start array *S*′ contains IDs of lexicographically sorted reads. (To obtain sorted reads, we use C++ std::sort, working in *O*(*mq* log *q*) time. Replacing it with radix sort we could reduce this time complexity to *O*(*mq*), yet it was not implemented.) In the initial phase we use array *S*′ to find reads with an overlap of length *m*, i.e., duplicates. If consecutive reads in array *S*′ are identical, then we mark in array *P* that the second one is suffix-overlapping the first. While traversing *S*′ we copy the reads without a suffix overlap to array *S* and the reads without a prefix overlap to array *Q*. The array *Q*′ is initially empty and *S*′ is flushed before the main loop. In each loop iteration we traverse the reads from array *S*, but in the order of their suffixes starting from position *i*+1. To this end, we need to store *σ* pointers for the current suffix in each group defined by the symbol at position *i*, which allows to find the minimal of the *σ* suffixes starting at the next symbol in *O*(*σ*) string comparisons. From now on, we assume that *σ* = *O*(1) for DNA, which allows to neglect the *σ* factor in the complexities. Note that finding the next read in *S* takes *O*(*σm*) = *O*(*m*) time, which gives *O*(*qm*) time for traversing once the whole array. “In parallel”, we also traverse the reads from array *Q* in their natural order. This resembles merging two sorted arrays (as used, e.g., in the textbook merge sort), with the difference that we do not sort the strings, but rather look for matches (overlaps) of length exactly *m* − *i*, in the *i*th iteration. Each check for an overlap takes *O*(*m*) time, hence a pass over the arrays of *S* and *Q* takes *O*(*qm*) time. Now, if for a read *x* ∈ *S* we find a suffix-overlapping read *y* ∈ *Q*, *y* ≠ *x*, we store this information in *P* together with the length of the overlap (i.e., *m* − *i*). If there is no overlap (of length *m* − *i*) for *x*, we copy the ID of *x* to array *S*′. Similarly, if while traversing *Q* we have not found any prefix-overlapping read for *y* ∈ *Q*, then we copy its ID to array *Q*′. When looking for overlaps we have to take care that the overlapped reads do not form a cycle. It is done by storing (in a separate auxiliary array) for each read that is not suffix-overlapped, the ID of the non-prefix-overlapped read in a chain of overlapped reads. For example, if there is a chain of overlapped reads *R*_1_ ∘ *R*_2_ ∘ *R*_3_ ∘ *R*_4_, we store for *R*_4_ that the “head” of the chain is *R*_1_. Then, when we look for a candidate for suffix overlap of *R*_4_, we can exclude *R*_1_. These data are easily updated in *O*(1) for each newly found overlap.

After a pass, *S*′ contains the IDs of only those reads which are not suffix-overlapped yet, sorted by their suffix starting at position *i*+1 and *Q*′ contains the IDs of only those reads which are not prefix-overlapped yet (in lexicographical order). The content of *S*′ and *Q*′ is then copied to *S* and *Q*, respectively. *S*′ and *Q*′ are flushed before the next iteration. (Of course, in a real implementation, the pointers to arrays are simply swapped, without physical array copying.) It can be easily noticed that the time complexity of the construction algorithm is *O*(*qm*(*m* + log *q*)). Using radix sort to initially sort the reads in the array *Q* would reduce the time complexity to *O*(*qm* ^2^).

Note that our current pseudogenome implementation does not handle reverse-complemented reads. Yet, our preliminary experiments with adding reverse-complemented reads to the generated sequence resulted in rather moderate improvement in the pseudogenome length (e.g., shorter by about 15%), while handling the queries requires significant changes in the used data structures (and possibly more space needed for them). For this reason, we leave this harder problem version as future work.

We note that this procedure is only a heuristic and does not guarantee to produce an optimal (shortest possible) pseudogenome. To see this, consider an example of three reads: *R*_1_ = *ACAT*, *R*_2_ = *CATG* and *R*_3_ = *ATCA*. According to the presented algorithm, we obtain the assembly (*R*_1_ ∘ *R*_2_)+*R*_3_ → *ACATGATCA* of length 9. Yet, the assembly (*R*_1_ ∘ *R*_3_) ∘ *R*_2_ → *ACATCATG* produces a sequence of length 8.

The actual pseudogenome representation depends on the given data (number of reads, read length etc.). In general it contains the *PG* string and the read array 𝓡^PG^ consisting of either 9- or 13-byte records. Consecutive records correspond to consecutive reads in the pseudogenome and contain the following fields:

- read offset in the pseudogenome (4 or 8 bytes, depending on the pseudogenome length),
- flag data occupying 1 byte (repetitive read flag, occurrence flag, single-occurrence flag, to be described later; several bits of this byte are not used),
- read index in the original read array 𝓡 (4 bytes).

Over the pseudogenome a search structure is built. Our basic solution is based on the classic suffix array (SA) [28], as a simple and fast full-text index. The *SA* ^PG^ elements require from 4 to 6 bytes. One element, associated with one pseudogenome suffix, stores the following fields:

- a read array index of the furthest read (of 𝓡^PG^) containing starting symbols of the given suffix (3 or 4 bytes, depending on the number of reads in the collection),
- start position of the suffix with regard to the beginning of the read (1 or 2 bytes, depending on the read length).

In order to access a suffix one has to obtain from the read array 𝓡^PG^ the offset of the specified read and add an offset of the suffix. Such organization enables straightforward identification of reads containing the sought prefix of the suffix.

Packing DNA symbols into bytes is a standard idea in compact data structures. We adopt this solution for the pseudogenome, in order to reduce the space use, minimize the rate of cache misses during searches and boost string comparisons (due to a lesser number of compared bytes on average). When the alphabet contains 4 symbols we handle the following compaction variants: (*i*) 2, 3 or 4 symbols per byte, (*ii*) 5 or 6 symbols per 2-byte unit (“short”). For the 5-symbol alphabet we pack either (*i*) 2 or 3 symbols per byte, or (*ii*) 4, 5 or 6 symbols per 2-byte unit.

Apart from the standard variant, we also implement a sparse suffix array (SpaSA) [8], which samples the suffixes in regular distances from the SA. The distances between sampled suffixes are specified by input parameter *s*. More precisely, if the pseudogenome is represented with *PG*[1…*p*] (w.l.o.g. assume that *s* divides *p*), the SpaSA index contains *p*/*s* suffix offsets: *s*, 2*s*, …, *p*. The data stored for a sampled suffix are like described above, plus *s* − 1 preceding symbols, in packed form. We set the *s* ≤ 6 limitation. Storing these *s* − 1 symbols allows not to access the pseudogenome sequence during a scan over the suffix array (cf. the *Q*3 query, described later) and is thus cache friendly. More precisely, the idea of storing the *s* − 1 symbols directly preceding a given suffix together with the corresponding offset in the sparse suffix array with sparsity *s* is to avoid verifying these symbols (of which some or all may belong to the query’s prefix of length at most *s* − 1) with an access into the pseudogenome, which resides in another array. In this way we have more local memory accesses. To make the current implementation easier and faster (due to less conditions necessary to check in the search procedure) the sparsity of the suffix array determines the packing of symbols, e.g., *s* = 5 means that 5 symbols are packed into 2-byte unit. Note that the *s* − 1 packed symbols require up to 2 bytes, hence the whole element for a suffix requires from 5 to 8 bytes.

For small values of *k* it is feasible to precompute all answers for the counting queries (*Q*2, *Q*4, and *Q*6). We assume the query is over the 4-symbol alphabet (ACGT). When the pseudogenome is small (up to 300 Mbases) we cache the answers for all *k* ≤ 10, and for larges pseudogenomes for all *k* ≤ 11. The *Q*2 and *Q*6 results occupy 4 bytes each and *Q*4 results 8 bytes. (Handling *Q*4 needs more space since *f* may appear in a single read several times.)

We note that the queries *Q*2, *Q*4, and *Q*6 are related. For example, the number of reads in which string *f* occurs only once (*Q*6) is often not much smaller than the total number of occurrences of *f* (*Q*4), and sometimes these values may be even equal; the equality of *Q*4 and *Q*6 also implies the same value of *Q*2. We make use of this fact and store answers also for *some* longer *k*-mers: up to *k* = 12 using 2-byte units and single bytes for *k* = 13. The precomputed answers are stored only if *Q*2 = *Q*4 = *Q*6, and *Q*2 less than 2^16^ − 1 or 2^8^ − 1, depending on the used variant. The opposite case is signaled on the 1- or 2-byte field with an unused value.

We call the main variant as variable-*k* PgSA. Still, our tool also has a fixed-*k* mode, in which the worst case complexities (although not significantly the performance on real data) improve. In this mode, after building the suffix array over the pseudogenome, the suffixes whose prefix of length *k* is not a substring of any read are removed from the SA. Such a check is performed for each suffix with a reference to 𝓡^PG^. Note that the removed suffixes may start only in reads which are overlapped by at most *k* − 2 symbols or are not overlapped at all. As each suffix in the found SA range contains at least one occurrence of the query *f*, the SA range width does not exceed ∣*Q*3∣.

Table 1 compares the worst-case time complexities for the queries *Q*1–*Q*7 of the existing algorithms. We use the notation ∣*Qx*∣ to represent the number of occurrences reported by query *Qx* (for *x* = 1, 3, 5, 7). In the following paragraphs we describe how the seven queries are performed in an order dictated by exposition clarity.

**Table 1:** Worst-case time complexities. To save space, the *O*(.) symbols around each formula were omitted. Note that *n* = *q*(*m* − *k*+1). The time complexities for PgSA are given for the fixed-*k* mode with SA sparsity set to 1. In the variable-*k* mode or when SA sparsity larger than 1 is used, the number of visited *SA* ^PG^ locations should be added to the PgSA complexities.

query	GkA (pos)	CGkA (pos)	GkA (seq)	CGkA (seq)	PgSA (pos/seq)
Q1	∣*Q*3∣	∣*Q*1∣ log log *n*	*k* log *n* + ∣*Q*3∣	*k* log σ + polylog *n* + |Q1| log log *n*	*k* log *p* + ∣*Q*3∣
Q2	∣*Q*3∣	log log *n*	*k* log *n* + ∣*Q*3∣	*k* log σ + polylog *n*	*k* log *p* + ∣*Q*3∣
Q3	∣*Q*3∣	∣*Q*3∣ log log *n*	*k* log *n* + ∣*Q*3∣	*k* log σ + polylog *n* + |Q3| log log	*k* log *p* + ∣*Q*3∣
Q4	1	log log *n*	*k* log *n*	*k* log σ + polylog *n*	*k* log *p* + ∣*Q*3∣
Q5	∣*Q*3∣	∣*Q*5∣ log log *n*	*k* log *n* + ∣*Q*3∣	*k* log σ + polylog *n* + |Q5| log log *n*	*k* log *p* + ∣*Q*3∣
Q6	∣*Q*3∣	log log *n*	*k* log *n* + ∣*Q*3∣	*k* log σ + polylog *n*	*k* log *p* + ∣*Q*3∣
Q7	∣*Q*3∣	∣*Q*7∣ log log *n*	*k* log *n* + ∣*Q*3∣	*k* log σ + polylog *n* + |Q7| log log *n*	*k* log *p* + ∣*Q*3∣

*Q*3 We binary search the suffix array *SA* ^PG^ for the string *f*, and for each potential match in the found range, pointing to some position in the pseudogenome *PG* (represented as a pair: read ID in the read array 𝓡^PG^ and the suffix offset with regard to the beginning of the read), we check in how many (0 or more) reads *f* really occurs. To this end, we check if the suffix offset shifted by *k* symbols does not exceed the read length *m*. If this is the case, we add its position to the output list, otherwise we terminate. Then, we scan over the read array 𝓡^PG^ backward, adding a position as long as the suffix offset plus *k* still does not exceed *m*. To speed up the binary search over *SA* ^PG^, we make use of a lookup table (LUT) storing the ranges of suffixes of all possible prefixes of length 11 (note that the number of LUT entries is, depending on the alphabet in a given dataset, 4^11^ or 5^11^, which is less than 50M).

*Q*4 We follow the procedure for *Q*3, but simply count the matches.

*Q*1 This query is related to *Q*3, but requires filtering, as *f* may occur in a read more than once. To this end, “occurrence flags” (stored in flag fields of 𝓡^PG^) are used. Initially, all these flags are set to false. During the iteration over reads (like in the *Q*3 query) only non-visited yet reads are added to the output list and for each new read the corresponding flag is set to true. The flag locations are also put on a stack, to remove them in *O*(∣*Q*1∣) time at the end, leaving all “occurrence flags” set to false in 𝓡^PG^. In general ∣*Q*1∣ ≤ ∣*Q*3∣, but since the equality often holds, we implemented some optimization. The array 𝓡^PG^ stores “repetitive read flag” for each read. This flag is true if the read contains at least one 11-mer at least twice. When we process the reads answering the *Q*1 query we verify the flag. If it is false we are sure that no *f* (of length at least 11) can appear in the read more than one time.

*Q*2 This query is to *Q*1 exactly like *Q*4 to *Q*3.

*Q*5 Again, this query is related to *Q*3, with extra filtration needed. Now “single-occurrence” flags in 𝓡^PG^ are used. The one-visit only mechanism for reads and unsetting the flags with aid of a stack is identical as in *Q*1. The operations on the stack take *O*(∣*Q*5∣) time, where ∣*Q*5∣ ≤ ∣*Q*3∣. Also here the “repetitive read” flags are used as a helpful heuristic.

*Q*6 This query is to *Q*5 exactly like *Q*4 to *Q*3, or *Q*2 to *Q*1.

*Q*7 We follow the procedure for *Q*5, only with replacing read IDs with the match positions.

As a final note, we admit that the flag fields stored in 𝓡^PG^ prevent multiple threads from querying the data structure concurrently, so the algorithm must be single-threaded. We are going to address this issue in a future version of the algorithm.

## Results

We ran experiments to confirm validity of our algorithm in practice. The testbed machine was equipped with an Intel i7 4930K 3.4 GHz CPU and 64 GB of RAM (DDR3-1600, CL11), running Linux 3.13.0-43-generic x86_64 (Ubuntu 14.04.1 LTS). Table 2 presents the datasets used in the tests. All these datasets are available at public repositories:

- E. coli (11.5M reads of 151 bp)—ftp://webdata:webdata@ussd-ftp.illumina.com/Data/SequencingRuns/MG1655/MiSeq_Ecoli_MG1655_110721_PF_R1.fastq.gz, ftp://webdata:webdata@ussd-ftp.illumina.com/Data/SequencingRuns/MG1655/MiSeq_Ecoli_MG1655_110721_PF_R2.fastq.gz, this dataset was used in the CGkA paper [29],
- GRCh37 (42.4M reads of 75 bp; no N symbols in the data)—http://crac.gforge.inria.fr/index.php?id = genomes-reads, this dataset was used in the CRAC paper [20],
- C. elegans (67.6M reads of 100 bp)—http://ftp.sra.ebi.ac.uk/vol1/fastq/SRR065/SRR065390/.

The command lines of the examined programs can be found in the PgSA package available at project homepage http://sun.aei.polsl.pl/pgsa.

**Table 2:** Dataset characteristics.

Dataset	No. reads [M]	Read length	Alphabet size	PG length [MB]
E. coli	11.5	151	5	551.4
GRCh37	42.4	75	4	567.9
C. elegans	67.6	100	5	1603.1

**Fig 2:**
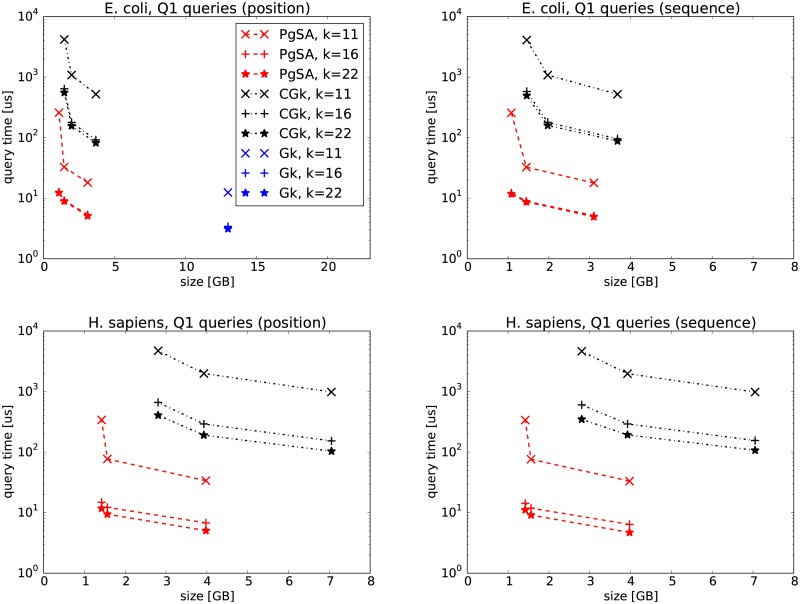
*Q*1 query results on E. coli (top row) and H. sapiens (bottom row) data. The three points in PgSA series correspond to sparsity *s* = 6 for the leftmost point, *s* = 3 (E. coli) or *s* = 4 (H. sapiens) for the middle point and *s* = 1 for the rightmost point. The three points in CGk series correspond to sampling rates *sr* of 512, 25 and 6 (E. coli), and 512, 22 and 6 (H. sapiens), respectively. On the left figures the query is given as a position in the read list, while on the right ones as a string.

**Fig 3:**
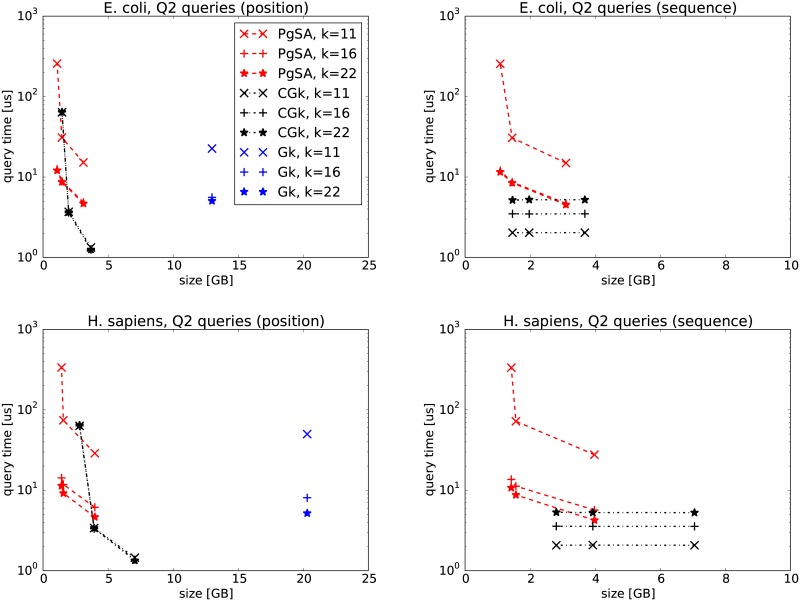
*Q*2 query results on E. coli (top row) and H. sapiens (bottom row) datasets. The three points in PgSA series correspond to sparsity *s* = 6 for the leftmost point, *s* = 3 (E. coli) or *s* = 4 (H. sapiens) for the middle point and *s* = 1 for the rightmost point. The three points in CGk series correspond to sampling rates *sr* of 512, 25 and 6 (E. coli), and 512, 22 and 6 (H. sapiens), respectively. On the left figures the query is given as a position in the read list, while on the right ones as a string.

**Fig 4:**
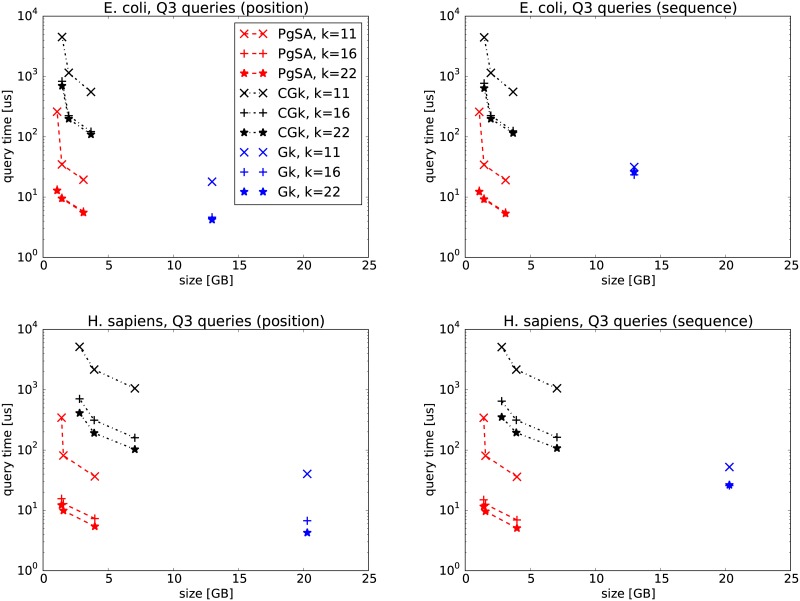
*Q*3 query results on E. coli (top row) and H. sapiens (bottom row) datasets. The three points in PgSA series correspond to sparsity *s* = 6 for the leftmost point, *s* = 3 (E. coli) or *s* = 4 (H. sapiens) for the middle point and *s* = 1 for the rightmost point. The three points in CGk series correspond to sampling rates *sr* of 512, 25 and 6 (E. coli), and 512, 22 and 6 (H. sapiens), respectively. On the left figures the query is given as a position in the read list, while on the right ones as a string.

**Fig 5:**
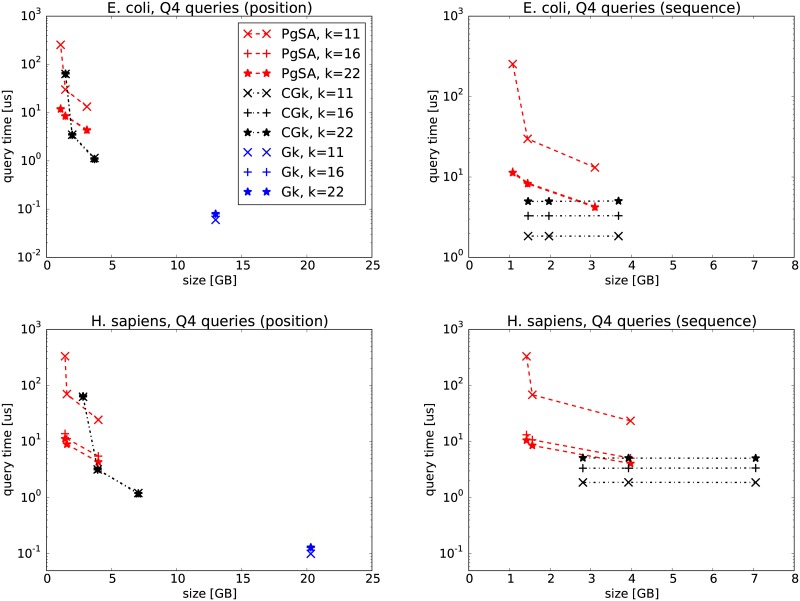
*Q*4 query results on E. coli (top row) and H. sapiens (bottom row) datasets. The three points in PgSA series correspond to sparsity *s* = 6 for the leftmost point, *s* = 3 (E. coli) or *s* = 4 (H. sapiens) for the middle point and *s* = 1 for the rightmost point. The three points in CGk series correspond to sampling rates *sr* of 512, 25 and 6 (E. coli), and 512, 22 and 6 (H. sapiens), respectively. On the left figures the query is given as a position in the read list, while on the right ones as a string.

In the first experiments, we compare PgSA versus GkA (version 2.1.0) and CGkA (version cgka_2013_08_21) on two datasets, E. coli and GRCh37-75bp-simulated reads (Figs 2, 3, 4, 5). We can see that in *Q*1 and *Q*3 queries PgSA is by more than an order of magnitude faster than CGkA at comparable or better compression rate. As expected, GkA is faster than CGkA (and sometimes faster, although not very significantly, than PgSA), yet requiring at least 3 times more space. The speed relation is different for *Q*2 and *Q*4 queries. Here CGkA defeats PgSA, sometimes by an order of magnitude. In the *Q*4 query, given by position, GkA is a clear winner in speed. We note that the tested (latest) GkA version (v2.1.0) does not support *Q*1, *Q*2 and *Q*4 when the query is given as a sequence rather than a position. Overall, we believe that PgSA offers attractive space-time tradeoffs for most queries, and in contrast to its competitors it handles arbitrary values of *k* (rather than a fixed one). Additionally, we note that the latest GkA and CGkA versions do not support the *Q*5–*Q*7 queries.

**Fig 6:**
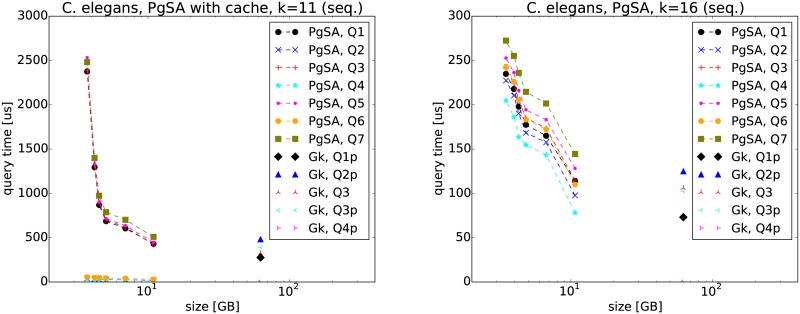
*Q*1–*Q*7 query results of PgSA and GkA on C. elegans dataset. The six points in the series correspond (from left to right) to sparsities *s* = 6, …, 1. The letter ‘p’ appended to some query names means that the query is given as a position in the read list.

In the next experiment we ran only PgSA and GkA on C. elegans dataset (Fig 6). We were not able to run CGkA on this dataset. The PgSA lines on the figures are for the queries *Q*1–*Q*7 given as a sequence (the time differences with regard to queries given as a position are up to 1 percent), and the left and right figure corresponds to the query length *k* = 11 and *k* = 16, respectively. Note that the results for the queries *Q*2, *Q*4, and *Q*6 are precomputed (cached) for *k* = 11.

In Tables 3 and 4 we detail out how much space is consumed by the components of the PgSA solution.

**Table 3:** E. coli dataset, space consumption. All sizes in megabytes.

SA sparsity	*PG*	𝓡^PG^	*SA* ^PG^	*LUT*	total
1	551	149	2205	195	3101
2	276	149	1378	195	1999
3	184	149	919	195	1447
4	276	149	689	195	1309
5	221	149	662	195	1227
6	184	149	551	195	1080

**Table 4:** C. elegans dataset, space consumption. All sizes in megabytes.

SA sparsity	*PG*	𝓡^PG^	*SA* ^PG^	*LUT*	total
1	1603	879	8016	195	10693
2	802	879	4809	195	6685
3	534	879	3206	195	4814
4	802	879	2405	195	4280
5	641	879	2244	195	3959
6	534	879	1870	195	3479

It may be informative to show the times and maximum memory usages for particular phases of the PgSA index construction. They are revealed in Table 5, for the variant based on the plain suffix array (i.e., sparsity *s* = 1). Morover, Table 6 contains index construction time, peak constructiontime memory usages and index spaces for the three solutions: GkArrays, CGk, and PgSA.

**Table 5:** Times and maximum memory usages for the PgSA index construction phases.

	Dataset		
	E. coli	GRCh37	C. elegans
**Maximal space usage [MB]**			
Pseudogenome (RAM)	1,361	2,193	5,258
Suffix array (HDD)	2,206	2,272	6,413
Total (RAM)	3,028	3,787	10,278
**Time [s]**			
Pseudogenome construction	189	219	603
Repetitive reads filter calculation	9	7	23
Suffix array construction	221	236	733
SA lookup construction	13	9	33
Total (including I/O)	452	509	1,476

**Table 6:** Index construction times and memory usages for the GkArrays, CGk and PgSA algorithms. For the CGkA algorithm *sr* denotes the sampling rate parameter, being a space-time tradeoff. CGkA crashed on the C. elegans dataset, in all tested configurations. GkA index is not written to disk, as opposed to the other two tools.

Index	Index space [MB]	Max. working space [MB]	User + system time [s]
**E. coli**			
GkA, *k* = 11	12,500	19,358	494
GkA, *k* = 22	12,400	17,881	439
CGkA (*sr* = 8), *k* = 11	3,120	3,858	1,171
CGkA (*sr* = 8), *k* = 22	3,120	3,859	1,235
CGkA (*sr* = 128), *k* = 11	1,538	3,857	1,128
CGkA (*sr* = 128), *k* = 22	1,538	3,859	1,181
PgSA (*s* = 1), var-k	3,101	3,028	452
PgSA (*s* = 2), var-k	1,999	1,951	394
PgSA (*s* = 3), var-k	1,447	1,411	344
**GRCh37**			
GkA, *k* = 11	21,300	32,887	844
GkA, *k* = 22	19,360	27,615	695
CGkA (*sr* = 8), *k* = 11	3,983	7,930	1,313
CGkA (*sr* = 8), *k* = 22	3,983	7,930	1,400
CGkA (*sr* = 128), *k* = 11	2,957	7,930	1,280
CGkA (*sr* = 128), *k* = 22	2,957	7,930	1,395
PgSA (*s* = 1), var-k	3,975	3,787	509
PgSA (*s* = 2), var-k	2,556	2,401	421
PgSA (*s* = 3), var-k	1,893	2,181	378
**C. elegans**			
GkA, *k* = 11	44,500	62,728	2,486
GkA, *k* = 22	39,300	62,740	2,295
PgSA (*s* = 1), var-k	10,693	10,278	1,476
PgSA (*s* = 2), var-k	6,685	6,364	1,275
PgSA (*s* = 3), var-k	4,814	5,134	1,065

Finally, we checked how replacing GkA with PgSA affects the CRAC performance (Table 7). We used CRAC v1.3.2 (http://crac.gforge.inria.fr) and the dataset GRCh37. Unfortunately, the build time grows several times (and even including the CRAC processing time the difference is at least by factor 2), yet the memory requirements of the PgSA-based variant are significantly lower, which may be a crucial benefit if a low-end workstation is only available.

**Table 7:** CRAC, *k* = 22, on the dataset GRCh37. Times in minutes, sizes in gigabytes.

Type	Build time	Build+CRAC time	Index size	Max mem. (build)	Max mem. (CRAC)
PgSA, *s* = 1	8.48	418.98	3.98	3.79	6.40
PgSA, *s* = 4	7.02	517.04	1.56	2.40	3.48
GkA	11.57	220.88	20.30	27.60	21.98

## Discussion

We proposed a new indexing structure for read collections. The experiments proved that this structure is much more compact than the existing solutions, GkA and CGkA. The running times of the counting queries are worse than of the CGkA, but in the listing queries PgSA is usually faster.

Several aspects of the presented scheme can be improved. We have noticed that using both direct and reverse-complemented reads in our construction of the pseudogenome reduces its size by about 15%. Still, this easy change for the construction is not equally easy to handle during the search, therefore the current implementation refrains from it. Additionally, our recent progress with read compression [32] suggests to build the pseudogenome from large datasets on disk (disk-based SA construction algorithms also exist, see, e.g., [33] and references therein). Finally, the sparse suffix array may be replaced by a recent sparse index, SamSAMi (sampled suffix array with minimizers) [34], with hopefully better performance.
